# Prevalence of Anxiety and Depression and Their Association with Health-Related Quality of Life Among Oncology Patients in Iraq: A Cross-Sectional Study

**DOI:** 10.3390/medicina62091652

**Published:** 2026-08-28

**Authors:** Ahmed Lateef Abed Alkhaqani, Miklós Sugár, Ammar Mahmood Jaber, Ali Abbas Rahi Al-Murshedi, Dávid Sipos, Nóra Rozmann, Bence Raposa

**Affiliations:** 1 Doctoral School of Health Sciences, Faculty of Health Sciences, University of Pécs, Vörösmarty Mihály Str. 4, 7621 Pécs, Hungary; miklos.sugar@etk.pte.hu (M.S.); jaber.ammar.mahmood@edu.pte.hu (A.M.J.); ali.abbas@edu.pte.hu (A.A.R.A.-M.); 2 Institute of Diagnostics, Faculty of Health Sciences, University of Pécs, Vörösmarty Mihály Str. 4, 7621 Pécs, Hungary; david.sipos@etk.pte.hu; 3 Institute of Emergency Care, Pedagogy of Health and Nursing Sciences, Faculty of Health Sciences, University of Pécs, Vörösmarty Mihály Str. 4, 7621 Pécs, Hungary; nora.rozmann@etk.pte.hu; 4 Institute of Basic Health Sciences, Health Visiting and Midwifery, Faculty of Health Sciences, University of Pécs, Vörösmarty Mihály Str. 4, 7621 Pécs, Hungary

**Keywords:** anxiety, depression, HRQoL, cancer, HADS, FACT-G, psychosocial

## Abstract

*Background and Objectives:* Anxiety and depression are among the most common psychological symptoms in patients with cancer and are associated with morbidity and impaired health-related quality of life (HRQoL), independent of the cancer stage. This study aimed to determine the prevalence of clinically significant anxiety and depression among adult oncology patients in Iraq and quantify the independent contribution of these symptoms to HRQoL. *Materials and Methods:* A cross-sectional study was conducted between 1 November and 15 December 2024, at the National Oncology Hospital in Najaf, Iraq. A total of 200 adult oncology patients were enrolled using consecutive sampling. Symptoms were measured using the Hospital Anxiety and Depression Scale (HADS), and HRQoL was measured using the Functional Assessment of Cancer Therapy-General (FACT-G). Descriptive statistics were used to summarize sample characteristics. Group comparisons were performed using Pearson’s chi-square test, the Fisher–Freeman–Halton exact test with Monte Carlo estimation, and one-way ANOVA, as appropriate. Associations among continuous variables were assessed using Pearson’s correlation and multiple linear regression. Statistical significance was set at *p* < 0.05. *Results:* The mean age (SD) was 52.2 (12.0) years, and 64.0% of the participants were male. Severe anxiety and severe depression were observed in 41.0% and 34.0% of the participants, respectively. Anxiety severity was significantly associated with age, sex, marital status, and treatment modality (all *p* < 0.01) and was significantly higher in females (58.3% vs. 31.2%) and unmarried patients. Depression severity was significantly associated with age (*p* = 0.005), sex (*p* < 0.001), and marital status (*p* < 0.001) but not with cancer stage (*p* = 0.223) or treatment modality (*p* = 0.530). The mean total FACT-G score was 58.2 (14.6), indicating low-to-moderate HRQoL. HADS-A and HADS-D were strongly intercorrelated (r = 0.741) and inversely correlated with FACT-G (r = −0.750 and r = −0.780, respectively; all *p* < 0.001). Multiple linear regression showed that higher HADS-A and HADS-D scores were independently associated with lower HRQoL, and the full seven-predictor model explained approximately 62% of the variance in FACT-G total scores (R^2^ = 0.62). *Conclusions:* Severe anxiety and severe depression were observed in 41.0% and 34.0% of participants, respectively. Higher anxiety and depression scores were independently associated with lower HRQoL.

## 1. Introduction

Cancer remains one of the leading global public health problems, with the number of survivors continuing to rise as a consequence of earlier diagnosis, advances in treatment, and population aging. It is currently the second largest cause of death worldwide [1]. The global cancer burden morbidity rate is also projected to increase over the next 20 years [2]. The diagnosis and treatment of cancer are potentially traumatic experiences that may result in many individuals experiencing serious psychopathology (e.g., anxiety and depression) across multiple cancer diagnosis groups, not only breast cancer, and this risk is not confined to any single tumor type [3]. The most common psychological problems for patients with cancer, regardless of the type of cancer, stage, or treatment, are depression and anxiety [4].

These symptoms range from mild emotional reactions to psychiatric disorders that produce functional impairment, reduced quality of life, treatment non-adherence, and higher cancer-related mortality [5]. Painful disease progression, physical disability, and lack of knowledge about the prognosis are closely correlated with high rates of depression, anxiety, and other mood disorders [6]. Previous studies have consistently identified anxiety and depression as the most frequent psychological symptoms among patients with cancer [7]. Concerning prevalence, it has been found that depression among cancer patients is about three times higher than that of the general population, and its prevalence has been reported to rise up to 71.8% [8]. The prevalence rates depend on the type of cancer, as more people with oropharyngeal cancer experience depression (22–57%) than those with pancreatic cancer counterparts (33–50%) [9].

Despite this substantial burden, anxiety and depression remain among the least recognized and least treated comorbidities in oncology [10,11]. The published literature also shows these inconsistencies. Although several studies have reported a higher prevalence of distress among women [12,13], a recent systematic review and meta-analysis observed an inverse pattern, with men reporting higher anxiety scores than women [14]. Estimates of stage-specific distress vary considerably, and the relative contribution of psychological symptoms to HRQoL, independent of sociodemographic and clinical variables, has not been quantified uniformly across different settings [15,16].

Recent studies from Middle Eastern oncology settings have reported a high burden of anxiety, depression, and impaired HRQoL; however, findings remain heterogeneous and are often derived from small, single-center samples [15,16]. Evidence from Iraq is particularly limited, and the independent associations of anxiety and depression with HRQoL have not been adequately quantified using validated instruments such as the HADS and FACT-G. Therefore, this study aimed to determine the prevalence of clinically significant anxiety and depression among adult oncology patients attending a tertiary cancer center in Iraq, examine their associations with sociodemographic and clinical characteristics, and evaluate their independent associations with HRQoL. By providing context-specific evidence from an underrepresented population, this study may inform the development of structured psychosocial screening and referral pathways in Iraqi oncology care.

## 2. Materials and Methods

### 2.1. Study Design

A cross-sectional design was employed to determine the prevalence of anxiety and depression, characterize their sociodemographic and clinical correlates, and evaluate their relationship with HRQoL in adult oncology patients. The design was selected as appropriate for the simultaneous estimation of symptom prevalence (descriptive) and assessment of associations among variables at a single time point.

### 2.2. Study Setting

The study was conducted at the National Oncology Hospital in Najaf, Iraq, a regional tertiary referral center that provides comprehensive inpatient and outpatient oncology services, including chemotherapy, radiotherapy, surgical oncology, and palliative care. The hospital has approximately 250 inpatient beds and serves approximately 2000 oncology outpatient visits per year, drawing patients from across the central and southern provinces of the country. The center was selected on objective grounds as one of the highest-volume oncology centers in Iraq, with a heterogeneous case mix (in terms of cancer type, stage, and treatment modality) that supported the recruitment of a representative sample of oncology patients.

### 2.3. Participants and Eligibility Criteria

The target population comprised adult patients (≥18 years) with a confirmed clinical diagnosis of any cancer type at any American Joint Committee on Cancer (AJCC) stage who were receiving active or post-treatment care at the participating centers. The inclusion criteria were as follows: (i) age ≥18 years; (ii) confirmed cancer diagnosis documented in the medical record; (iii) sufficient cognitive and linguistic capacity to provide informed consent and complete a 15–20 min structured interview; and (iv) willingness to participate. The exclusion criteria were as follows: (i) severe cognitive impairment, delirium, or a documented psychiatric disorder unrelated to cancer that would interfere with self-report; (ii) a clinically unstable medical condition or severe pain or fatigue precluding completion of the interview; and (iii) communication or hearing barriers that were not resolvable in the clinical setting.

### 2.4. Sampling and Sample Size

Consecutive sampling was used: every eligible patient attending the oncology outpatient and inpatient services on the data collection days was approached in sequence until the target sample was reached. An a priori sample size estimate was calculated for a finite source population of approximately 2000 patients using OpenEpi version 3.01 (OpenEpi, open-source web application; https://www.openepi.com/; accessed on 21 March 2026), applying a single-proportion formula with an anticipated psychological distress prevalence of 80% (based on regional oncology HRQoL studies), a precision of ±5%, and a 95% confidence interval, yielding a minimum required sample size of 200 participants. Of the 240 patients approached, 200 completed the questionnaire (response rate: 83.3%), exceeding the minimum estimate.

### 2.5. Study Instruments

Data were collected using a structured questionnaire comprising three components: a demographic and clinical data form, the Hospital Anxiety and Depression Scale (HADS), and the Functional Assessment of Cancer Therapy–General (FACT-G).

#### 2.5.1. Demographic and Clinical Data Form

This investigator-developed form was constructed based on variables consistently identified in the oncology psychosocial literature as predictors of distress and HRQoL. It captured age, sex, marital status, education level, occupation, cancer type, cancer stage (I–IV, classified according to the AJCC TNM staging system as recorded in the patient’s medical chart), treatment modality, and treatment duration. “Perception of cancer” was operationalised as the patient’s self-reported appraisal of their illness as “manageable” versus “threatening,” recorded on a single direct item. Patients receiving multiple modalities concurrently were categorized as receiving “combination therapy” (most commonly chemotherapy combined with radiotherapy or surgery); mono-modality categories (chemotherapy alone, radiotherapy alone, and surgery alone) were recorded only when the patient was receiving that single modality at the time of the interview.

#### 2.5.2. Hospital Anxiety and Depression Scale (HADS)

The HADS, originally developed by Zigmond and Snaith [17], is a 14-item self-report screening tool widely used in oncology because it deliberately excludes somatic items (e.g., fatigue, sleep disturbance, and weight loss) that overlap with cancer- and treatment-related symptoms, thereby reducing diagnostic confounding [18]. It comprises two seven-item subscales—anxiety (HADS-A) and depression (HADS-D)–each rated 0–3, yielding subscale scores of 0–21. Following the cut-offs validated in oncology populations and confirmed in Arabic translations of the scale, scores of 0–7 were classified as (normal), 8–15 as borderline (mild-to-moderate), and 16–21 as clinically significant (severe) scores. This three-level classification is used to describe overall symptom burden in the sample. For bivariate association analyses with sociodemographic and clinical variables, the normal and borderline categories were combined a priori into a single ‘non-severe’ category and compared against the ‘severe’ category, to ensure adequate expected cell counts for χ^2^/Fisher’s exact testing and to align with the clinically actionable referral threshold. In the present sample, Cronbach’s α was 0.86 for HADS-A and 0.84 for HADS-D, indicating a good internal consistency.

#### 2.5.3. Functional Assessment of Cancer Therapy–General (FACT-G)

The FACT-G, in its 27-item version 4, assesses four dimensions of well-being: physical (PWB, 7 items, score range 0–28), social/family (SWB, 7 items, score range 0–28), emotional (EWB, 6 items, score range 0–24), and functional (FWB, 7 items, score range 0–28) well-being. Items are rated on a 5-point Likert scale from 0 (“not at all”) to 4 (“very much”), with subscale and total scores summed such that higher values reflect better HRQoL; the total score ranges from 0 to 108. The instrument was selected for its widespread use in oncology, established reliability, and sensitivity to clinically relevant changes [19]. In the present sample, Cronbach’s α was 0.91 for the total FACT-G, with subscale alphas ranging from 0.78 (EWB) to 0.87 (PWB).

### 2.6. Data Collection

Data were collected between 1 November and 15 December 2024, through structured face-to-face interviews conducted in a private room within the hospital outpatient clinic. Interviews were performed by two trained oncology nurses who completed a half-day standardization session with the principal investigator, covering an item-by-item review of the questionnaire, neutral phrasing of probes, and protocols for managing emotional distress during the interview. To minimize interviewer bias, both interviewers used a standardized script and recorded the responses verbatim. To control for variations in symptom burden across chemotherapy cycles, interviews with patients receiving chemotherapy were conducted on the day of the scheduled visit, before treatment administration, rather than immediately after infusion. Each interview lasted approximately 15–20 min. If a participant became visibly fatigued or distressed, the interview was paused, the participant was offered rest, water, or rescheduling to a second visit, and reminded of their right to withdraw without consequence. Patients who were unable to complete the interview after such accommodation were withdrawn from the sample, and the principal investigator was notified to coordinate clinical follow-up when appropriate.

### 2.7. Statistical Analysis

Data were analyzed using IBM SPSS Statistics for Windows, version 26.0 (IBM Corp., Armonk, NY, USA). The revised contingency-table analyses reported below were independently recalculated from the original observed cell counts using SciPy version 1.17.0.

Descriptive statistics were used to summarize the sociodemographic, clinical, and outcome variables. Categorical variables are presented as frequencies and percentages, whereas continuous variables are reported as means and standard deviations. The Shapiro–Wilk test was used to assess the normality of continuous variables, and Levene’s test was used to evaluate the homogeneity of variances before parametric group comparisons.

For descriptive purposes, HADS-A and HADS-D scores were classified using the conventional three-category structure: normal (0–7), borderline (8–15), and severe (16–21). For the inferential analyses reported below, HADS-A and HADS-D scores were dichotomized as non-severe (0–15) or severe (16–21). All observed counts, row percentages, and *p*-values reported in these tables were calculated from the same two-category outcomes.

Pearson’s chi-square test was used to examine associations between categorical variables when the expected cell-frequency assumptions were satisfied. For sparse contingency tables, the Fisher–Freeman–Halton exact test was estimated using Monte Carlo simulation with 1,000,000 samples. Accordingly, Pearson’s chi-square test was used for the analyses of age group and sex, whereas exact testing with Monte Carlo estimation was applied where sparse cells affected the contingency tables for marital status, cancer stage, or treatment modality.

One-way analysis of variance (ANOVA) was used to compare mean HADS-A, HADS-D, and FACT-G scores across AJCC cancer-stage groups. When the omnibus ANOVA result was statistically significant, Tukey’s honestly significant difference post hoc test was used for pairwise comparisons. Pearson’s correlation coefficient (r) was used to quantify the relationships between HADS-A, HADS-D, and FACT-G domain and total scores.

A multiple linear regression model was constructed using the simultaneous forced-entry method. The total FACT-G score was specified as the dependent variable, while HADS-A, HADS-D, age, sex, marital status, cancer stage, and treatment modality were specified a priori as independent variables. All seven predictors were entered simultaneously into the model in a single block rather than being selected or entered sequentially. Sex was coded as 0 = female and 1 = male. Marital status was dichotomized as 0 = married and 1 = single, widowed, or divorced. Treatment modality was dichotomized as 0 = chemotherapy alone and 1 = no active treatment or another treatment modality, including combination therapy, radiotherapy, or surgery. Cancer stage was entered as an ordered clinical variable from Stage I = 1 to Stage IV = 4. Multicollinearity was assessed using variance inflation factors.

All statistical tests were two-tailed, and *p*-values below 0.05 were considered statistically significant. Exact *p*-values are reported to three decimal places, while values below 0.001 are presented as *p* < 0.001.

### 2.8. Ethical Considerations

Administrative permission and ethical approval were obtained before data collection from the Iraqi Ministry of Health, Najaf Health Directorate, Training and Human Development Center, and Director of the National Oncology Hospital (approval No. 37706, dated 1 November 2024). This study was conducted in accordance with the principles of the Declaration of Helsinki. Written informed consent was obtained from all participants prior to enrolment. No personal identifiers were collected in the research dataset, and participants were free to refuse to participate or withdraw at any time without consequences for their clinical care.

## 3. Results

### 3.1. Sociodemographic and Clinical Characteristics

A total of 200 patients with cancer participated in this study. The sociodemographic and clinical characteristics of the sample are presented in Table 1. The mean age (SD) was 52.2 (12.0) years, with the largest age group being 45–59 years (34.0%), followed by 30–44 years (25.0%) and ≥60 years (20.0%). Male participants predominated (64.0%), and the majority were married (62.0%) with primary-level education or below (64.0% combined illiterate and primary education). Most participants (78.0%) were unemployed or homemakers.

Cancer staging was based on the AJCC TNM classification recorded in medical charts. The most common cancers were lung (16.0%), prostate (13.0%), breast (13.0%), and gastrointestinal tract (13.0%); the remaining 27.0% comprised a heterogeneous group of less frequent diagnoses (referred to as “Other”), including head and neck, lymphoma, ovarian, cervical, brain, and unspecified solid tumors. Stage II disease predominated (49.0%), followed by Stage III (28.0%), Stage I (19.0%), and Stage IV (4.0%). Approximately half of the participants (46.0%) were receiving chemotherapy as a single modality; 40.0% were receiving combination therapy (most commonly chemotherapy with radiotherapy or surgery); 6.0% were receiving radiotherapy only; 2.0% were receiving surgery only; and 6.0% were not receiving active treatment. The mean total treatment duration was 17.31 ± 14.98 months.

### 3.2. Distribution of Anxiety and Depression Severity

**Figure 1:**
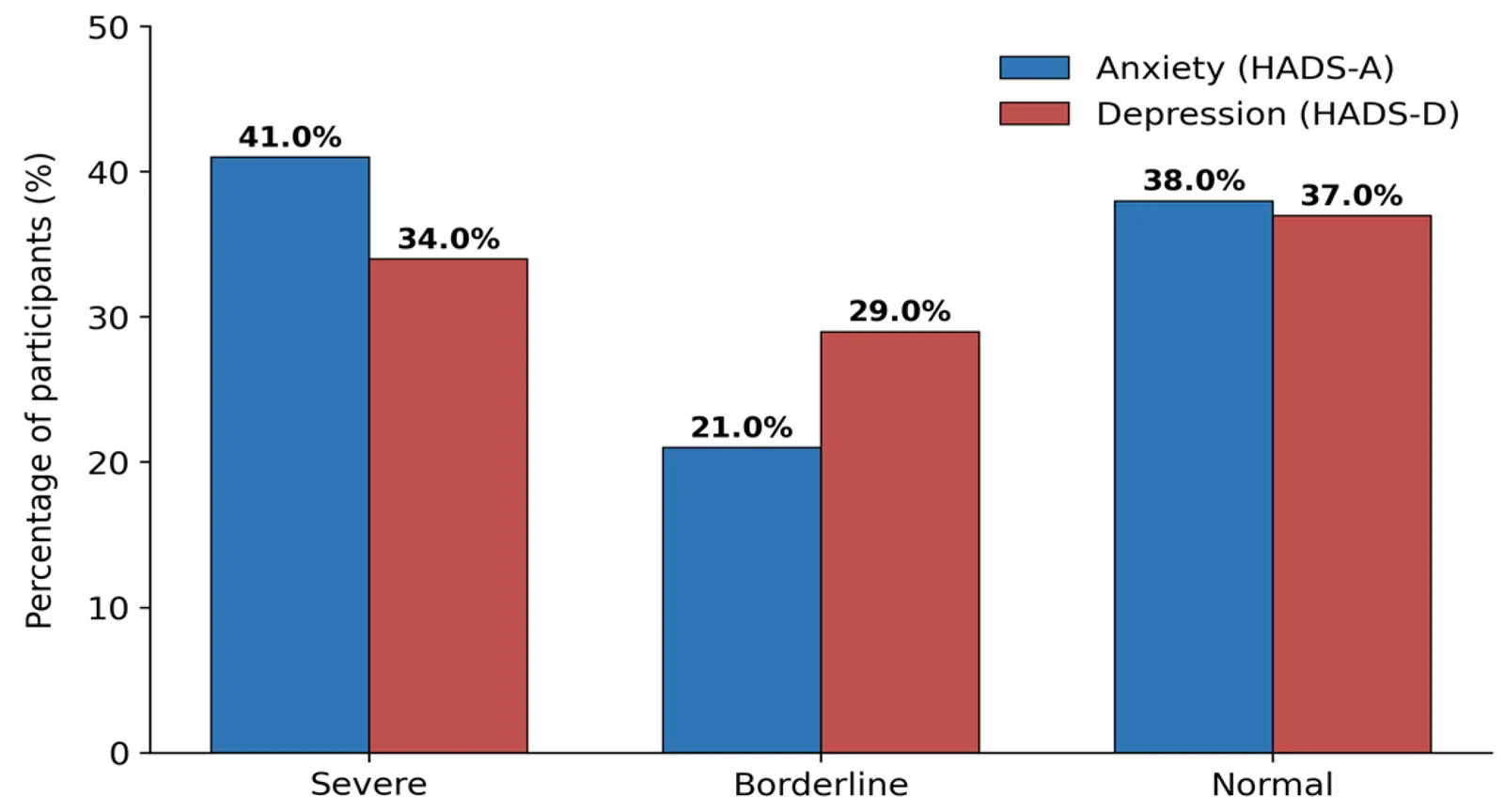
Distribution of HADS anxiety and depression severity categories in the total study sample. HADS-A (Hospital Anxiety and Depression Scale—Anxiety) and HADS-D (Hospital Anxiety and Depression Scale—Depression) subscale scores range from 0 to 21 and were descriptively classified as normal (0–7), borderline (8–15), or severe (16–21).

Based on the conventional three-category HADS classification, 38.0% of participants had normal anxiety scores, 21.0% had borderline anxiety scores, and 41.0% had severe anxiety scores. For depression, 37.0% had normal scores, 29.0% had borderline scores, and 34.0% had severe scores. Thus, severe anxiety and severe depression were observed in 41.0% and 34.0% of participants, respectively (Figure 1).

### 3.3. Health-Related Quality of Life

**Figure 2:**
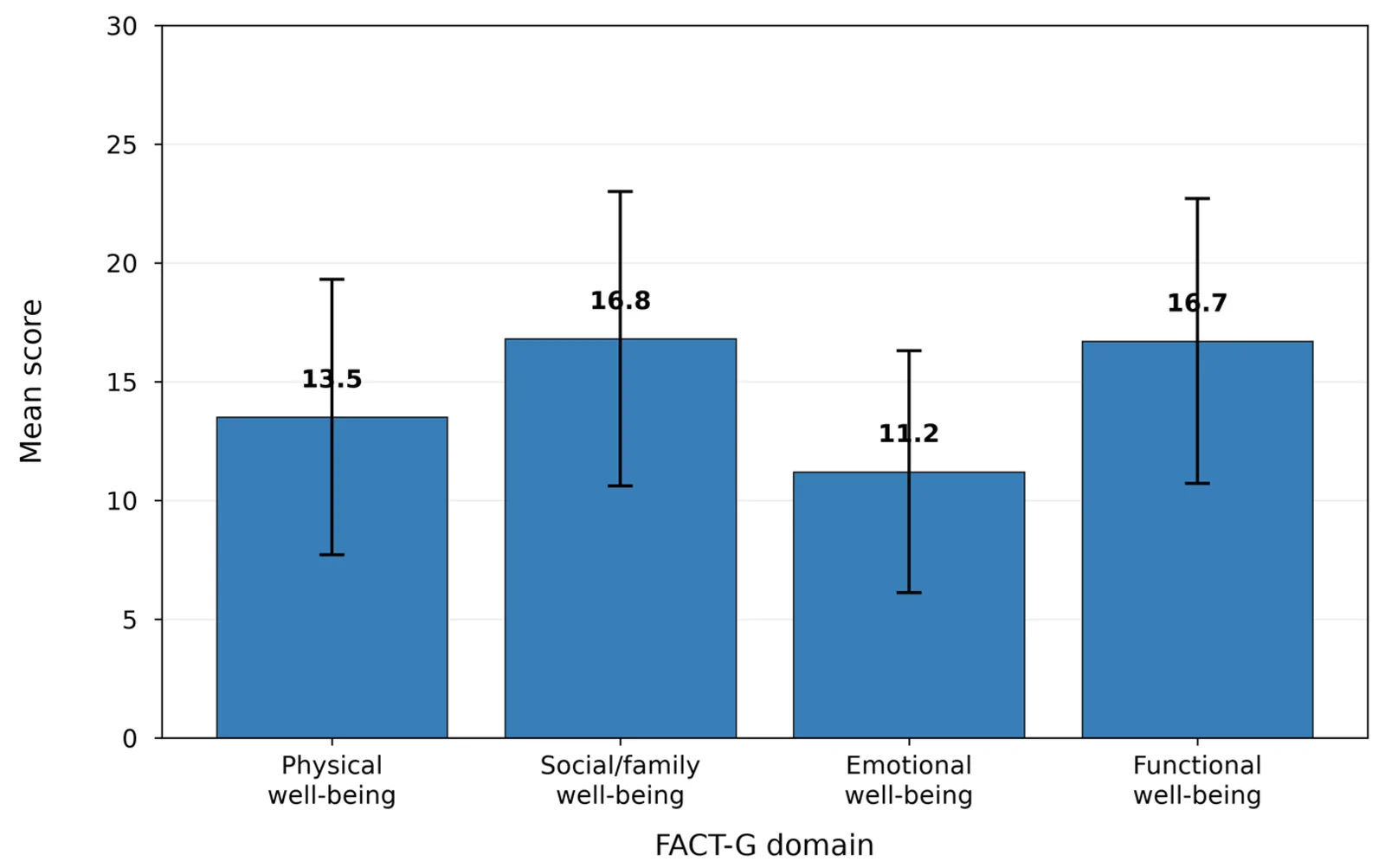
Mean Functional Assessment of Cancer Therapy—General (FACT-G) domain scores among oncology patients. Physical, social/family, and functional well-being subscales range from 0 to 28; emotional well-being ranges from 0 to 24. Higher scores indicate better HRQoL.

The mean total FACT-G score was 58.2 (14.6) (possible range, 0–108), indicating low-to-moderate HRQoL. The mean values were lowest for physical well-being (13.5 ± 5.8) and emotional well-being (11.2 ± 5.1) and higher for social/family well-being (16.8 ± 6.2) and functional well-being (16.7 ± 6.0), indicating that the physical and emotional domains were the principal contributors to HRQoL impairment in this sample (Figure 2).

### 3.4. Psychological Distress and HRQoL by Cancer Stage

**Figure 3:**
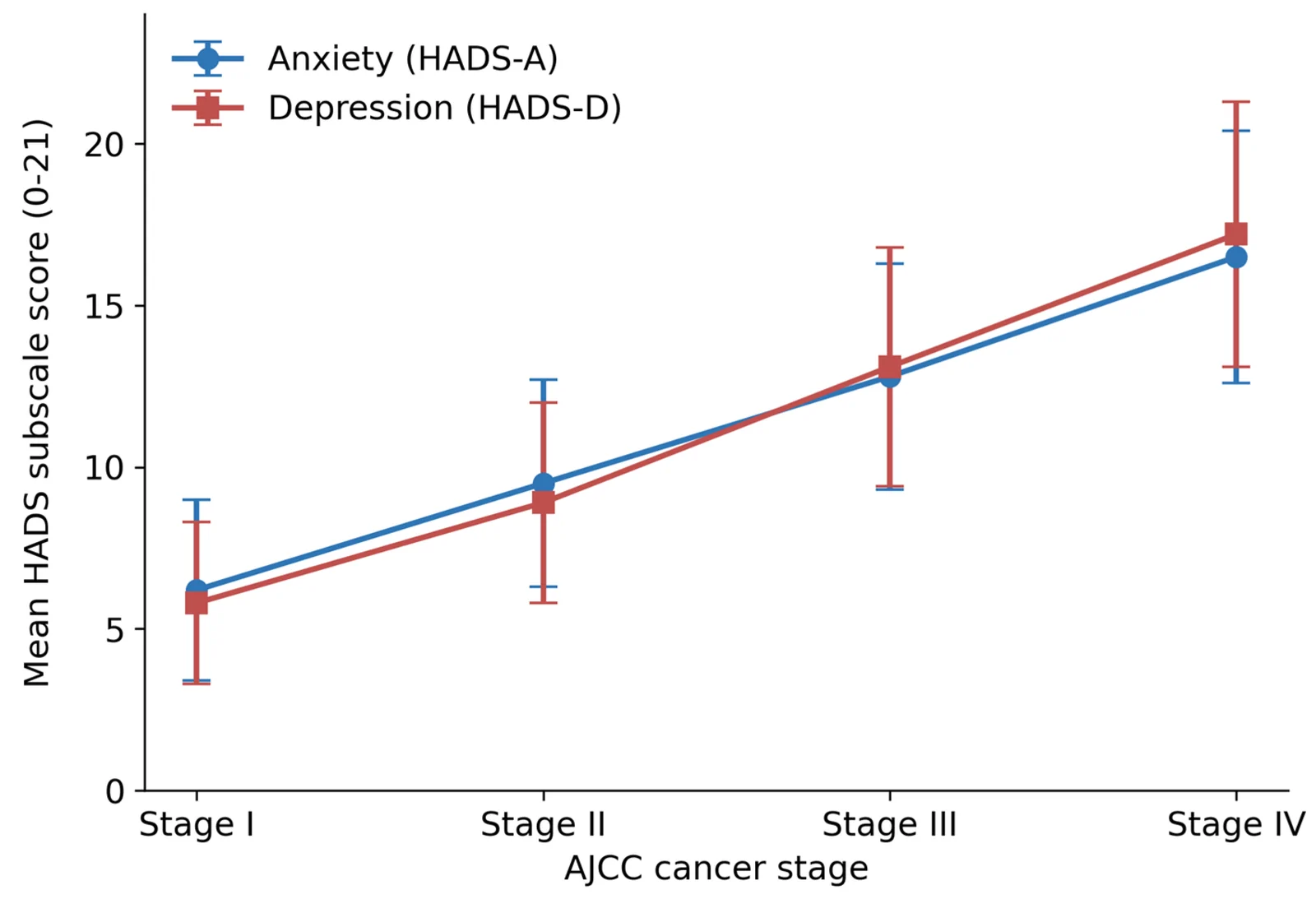
Psychological distress across American Joint Committee on Cancer (AJCC) stages. Hospital Anxiety and Depression Scale—Anxiety (HADS-A) and Hospital Anxiety and Depression Scale—Depression (HADS-D) subscale scores range from 0 to 21;higher scores indicate greater symptom severity.

**Figure 4:**
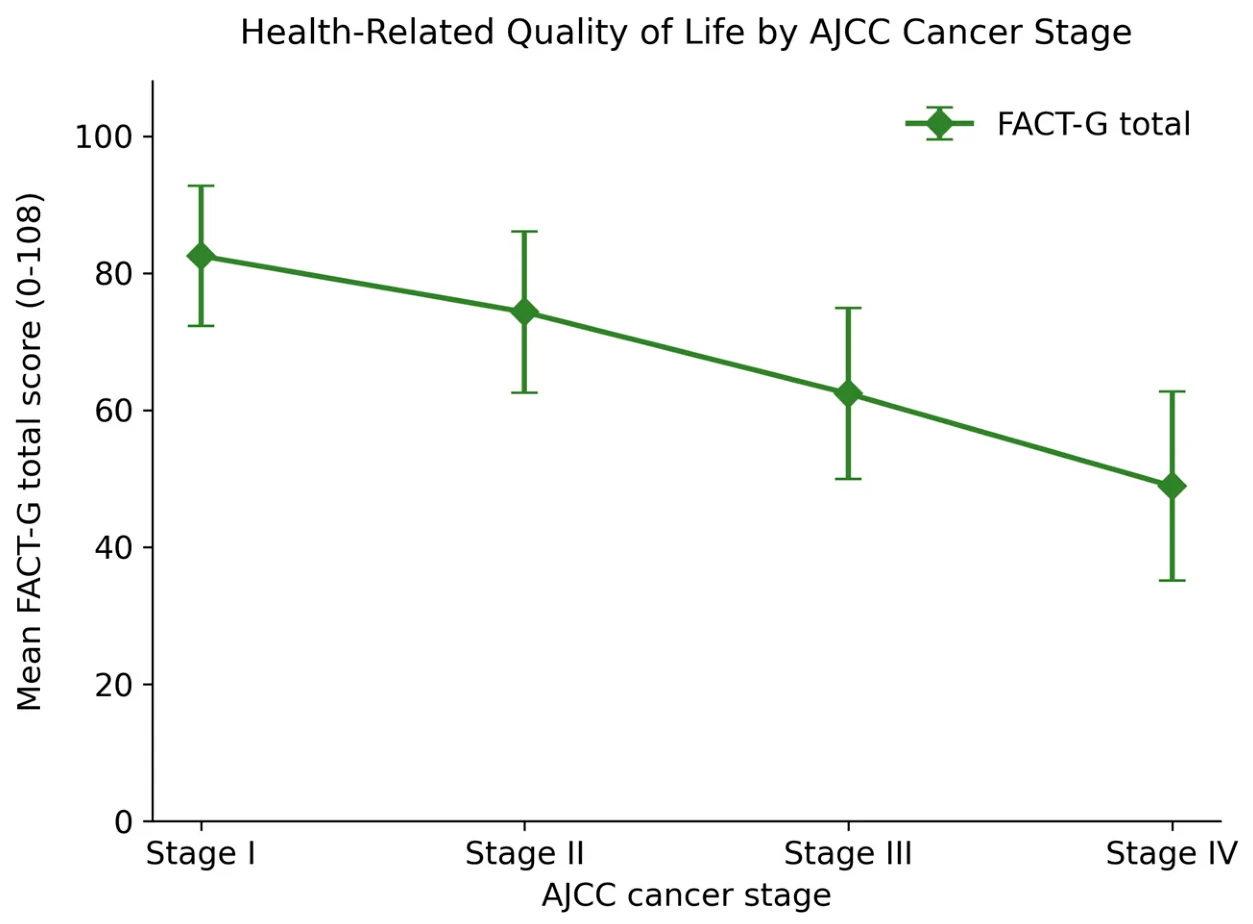
Mean Functional Assessment of Cancer Therapy—General (FACT-G) total score by American Joint Committee on Cancer (AJCC) stage. The FACT-G total score ranges from 0 to 108; higher scores indicate better HRQoL.

Mean HADS-A, HADS-D, and total FACT-G scores differed significantly across AJCC stages on one-way ANOVA (all *p* < 0.001; Table 2). Anxiety and depression scores increased monotonically across stages, whereas the HRQoL declined. Stage IV patients exhibited the highest distress (HADS-A 16.5 ± 3.9; HADS-D 17.2 ± 4.1) and the lowest HRQoL (FACT-G 48.9 ± 13.8). Tukey HSD post hoc tests indicated that Stages III and IV differed significantly from Stages I and II for all three outcomes (*p* < 0.05). These stage-specific patterns are illustrated in Figure 3 and Figure 4.

### 3.5. Sociodemographic and Clinical Correlates of Anxiety and Depression Severity

Chi-square and exact tests applied to the dichotomized HADS severity outcomes showed that severe anxiety was significantly associated with age group (*p* < 0.001), sex (*p* < 0.001), marital status (*p* < 0.001), and treatment modality (*p* = 0.002), but not with cancer stage (*p* = 0.626; Table 3). Severe depression was significantly associated with age group (*p* = 0.005), sex (*p* < 0.001), and marital status (*p* < 0.001), whereas no significant associations were observed with cancer stage (*p* = 0.223) or treatment modality (*p* = 0.530; Table 3). The proportions of severe anxiety and depression were higher among younger participants, women, and unmarried participants. Severe anxiety was also particularly frequent among participants who were not receiving active treatment.

### 3.6. Correlations and Multivariable Regression

Anxiety and depression scores were strongly intercorrelated (HADS-A with HADS-D: r = 0.741, *p* < 0.001) and both were strongly inversely correlated with the total FACT-G score (HADS-A: r = −0.750; HADS-D: r = −0.780; both *p* < 0.001) and with each FACT-G domain (all *p* < 0.001; Table 4).

In the multiple linear regression model (Table 5), after adjusting for age, sex, marital status, cancer stage, and treatment modality, HADS-A (β = −0.34, *p* < 0.001) and HADS-D (β = −0.41, *p* < 0.001) remained independent predictors of HRQoL. The full seven-predictor model explained approximately 62% of the variance in FACT-G total scores (R^2^ = 0.62). These findings indicate that anxiety and depression were independently associated with lower HRQoL after adjustment for the other variables included in the model.

## 4. Discussion

### 4.1. Prevalence of Anxiety and Depression

In this cross-sectional study, severe anxiety and severe depression were observed in 41.0% and 34.0% of participants, respectively, indicating a substantial burden of psychological distress. These findings are broadly consistent with reports from other Middle Eastern oncology populations, although direct comparisons should be made cautiously because studies differ in cancer composition, disease stage, sampling procedures, assessment instruments, and HADS thresholds [15,16]. The strong inverse correlations between HADS-A, HADS-D, and FACT-G scores further demonstrate the clinical relevance of psychological distress in this population and are consistent with previous evidence linking anxiety and depression to impaired HRQoL among patients with cancer [20]. Nevertheless, because the present study was cross-sectional and conducted at a single tertiary oncology center, these prevalence estimates should not be interpreted as nationally representative figures for the Iraqi oncology population [21].

### 4.2. Sociodemographic and Clinical Correlates of Psychological Distress

Severe anxiety and depression were more frequent among younger participants, women, and unmarried participants. Severe anxiety was observed in 58.3% of women compared with 31.2% of men, while severe depression was observed in 50.0% of women compared with 25.0% of men. These findings are consistent with previous oncology studies identifying female sex as an important correlate of anxiety and depression [12,13], although they contrast with the meta-analysis by Vitale et al. (2024), which reported higher anxiety scores among men [14]. This divergence may reflect differences in study populations, cancer characteristics, cultural contexts, symptom-reporting patterns, or analytical methods.

Marital status was also associated with both anxiety and depression severity. Severe symptoms were generally more frequent among single, widowed, or divorced participants than among married participants, suggesting that the presence of a partner may be associated with greater perceived emotional or practical support [22]. However, marital status should not be interpreted as a direct measure of social support, and the cross-sectional design does not permit conclusions regarding the direction or mechanisms of this association. Severe anxiety was additionally associated with treatment modality and was particularly frequent among participants who were not receiving active treatment. In contrast, neither cancer stage nor treatment modality was significantly associated with dichotomized depression severity.

### 4.3. Independent Association of Psychological Distress with HRQoL

Anxiety and depression were strongly and inversely correlated with HRQoL. Higher HADS-A and HADS-D scores were associated with lower FACT-G total and domain scores, indicating that greater psychological symptom severity was accompanied by poorer physical, emotional, social, and functional well-being. These findings are consistent with previous oncology research demonstrating a close relationship between psychological distress and impaired HRQoL [20,23,24,25,26].

In the multivariable analysis, HADS-A and HADS-D remained significantly associated with lower FACT-G total scores after adjustment for age, sex, marital status, cancer stage, and treatment modality. Depression showed the larger standardized association with HRQoL. The full seven-predictor model explained approximately 62% of the variance in FACT-G total scores, suggesting that psychological and clinical factors together accounted for a substantial proportion of the observed variation in HRQoL. Because HADS-A and HADS-D were entered simultaneously with the sociodemographic and clinical covariates, their significant coefficients indicate associations with HRQoL that were independent of the other variables included in the model.

Cancer stage was also independently associated with FACT-G total scores, and HRQoL declined progressively across the cancer-stage groups. This finding suggests that psychological distress and disease severity may make distinct contributions to patients’ perceived quality of life. Nevertheless, the cross-sectional design does not permit causal inference; therefore, anxiety and depression should be interpreted as clinically relevant correlates of HRQoL rather than proven causes of its deterioration.

### 4.4. Implications for Psychosocial Care in Iraq

These findings carry direct implications for clinical practice at the study center and comparable tertiary oncology settings in Iraq. Currently, the National Oncology Hospital in Najaf does not operate a structured, protocol-driven psycho-oncology referral pathway; patients identified as being in psychological distress during routine clinical encounters are referred on an ad hoc basis to general psychiatric services outside the oncology department without a standardized screening trigger, dedicated psycho-oncology staff, or systematic follow-up. Given that severe anxiety and severe depression were observed in 41.0% and 34.0% of participants, respectively, this ad hoc model may fail to identify and support a substantial proportion of patients with clinically relevant psychological needs.

The results support three concrete, resource-conscious priorities for the study center and comparable Iraqi oncology settings. First, brief validated instruments, such as the HADS, could be integrated into routine oncology visits as a low-cost, low-burden screening trigger, given their demonstrated feasibility, acceptability, and internal consistency in the present sample. Second, family- and caregiver-inclusive counselling models may be particularly well-suited to this cultural context, where family and community networks are typically central to patient support. Third, formal referral agreements between the oncology department and existing psychiatric or clinical psychology services, even where dedicated psycho-oncology staff are not yet available, would allow at-risk patients identified through routine screening to be systematically triaged rather than being referred inconsistently.

The principal barriers to implementing structured psychosocial screening and referral in this setting are likely to include the absence of dedicated psycho-oncology staff, limited clinician time during high-volume outpatient visits, the stigma historically associated with psychiatric referral in this cultural context, and the absence of a formal reimbursement or resource pathway for psychosocial services within Iraqi oncology care. Addressing these barriers will require institutional investment and, plausibly, multicenter advocacy beyond the scope of a single-center study. Nonetheless, the magnitude and independence of the distress–HRQoL association observed in this study provide a quantitative, locally generated justification for prioritizing this investment [27,28].

### 4.5. Strengths and Limitations

This study had several strengths. It used internationally recognized, oncology-validated instruments (HADS and FACT-G) with good internal consistency in the present sample (Cronbach’s α 0.84–0.91), employed an explicitly defined a priori sampling and analytic framework, and reported analyses using both continuous scores and dichotomized outcome variables. The single-center setting of a high-volume tertiary oncology hospital provided a heterogeneous case mix and a well-defined denominator population. The use of structured interviews conducted by trained oncology nurses, rather than self-completed paper questionnaires, reduced item non-response and accommodated participants with low literacy levels.

However, this study had several limitations. The single-center design of one tertiary referral hospital in southern Iraq may limit the generalizability of the findings to other healthcare systems and cultural contexts. The use of self-report instruments is inherently subject to response bias, including socially desirable responses, reluctance to disclose psychological symptoms in a culturally conservative setting, and the influence of acute clinical status (e.g., fatigue and pain) on responses. These effects were mitigated but not eliminated through interviewer training and pre-infusion scheduling of chemotherapy interviews. Fifth, the cross-sectional design precludes inferences regarding the direction or temporal evolution of observed associations. Finally, although a priori predictors were specified for the regression model, residual confounding by unmeasured factors, including socioeconomic status, prior psychiatric history, and quality of family support cannot be excluded.

Third, the sample size calculation was based on an anticipated psychological distress prevalence of 80%, derived from regional oncology HRQoL studies [15,16]; however, the observed prevalence of severe anxiety (41.0%) and severe depression (34.0%) was notably lower. This discrepancy suggests that the a priori prevalence estimate may have been inflated, likely due to heterogeneity across regional studies, differences in distress measurement instruments, and the specific clinical and cultural contexts of the Iraqi population. As a result, the study may have been underpowered to detect smaller effect sizes in subgroup comparisons, and the 95% confidence interval around the prevalence estimates was wider than would have been achieved with a sample size >300. Future multicenter studies with larger sample sizes are warranted to refine prevalence estimates and increase precision.

## 5. Conclusions

Severe anxiety and severe depression were observed in 41.0% and 34.0% of participants, respectively. Higher anxiety and depression scores were independently associated with lower HRQoL, while the full seven-predictor model explained approximately 62% of the variance in FACT-G total scores. The burden of psychological distress was particularly pronounced among women, unmarried patients, and those with advanced-stage disease. These findings identify anxiety and depression as critical, modifiable determinants of patient-reported outcomes in oncology and provide a quantitative basis for routine psychological screening using brief validated instruments, such as the HADS, within multidisciplinary cancer services in the future. The integration of evidence-based psychosocial intervention, structured psychotherapy, peer support programs, and judicious pharmacological treatment should be considered a standard, rather than an ancillary, component of oncology care in similar settings. Future prospective multicenter research is warranted to evaluate the comparative effectiveness of these strategies on distress and HRQoL trajectories across the cancer continuum.

At the study center, psychological services are available on an ad hoc referral basis; however, routine distress screening has not yet been integrated into standard oncology care. Patients with severe symptoms may be referred to on-site psychiatry or counseling services; however, barriers include the limited availability of trained psycho-oncologists, cultural stigma associated with mental health care, lack of standardized screening protocols, and high patient volume relative to staff capacity. To improve care, priorities include the following: (1) implementation of routine HADS or similar brief screening at initial oncology consultation and during treatment transitions; (2) training oncology nurses in basic psychosocial assessment and supportive communication; (3) establishing structured referral pathways to psychiatry, psychology, and peer support programs; and (4) addressing cultural barriers through patient and family education on the normality and treatability of cancer-related distress. These steps are feasible within the existing infrastructure and would align Iraqi oncology care with international psychosocial care standards.

## Figures and Tables

**Table 1:** Sociodemographic and clinical characteristics of the study sample.

Variable	Category	Frequency (*n*)	Percent (%)
Age group	<30 years	42	21.0
	30–44 years	50	25.0
	45–59 years	68	34.0
	≥60 years	40	20.0
	Mean (SD): 52.2 ± 12.0 years		
Sex	Male	128	64.0
	Female	72	36.0
Marital status	Single	40	20.0
	Married	124	62.0
	Widowed	12	6.0
	Divorced	24	12.0
Education	Illiterate	48	24.0
	Primary	80	40.0
	Secondary (high school)	28	14.0
	Diploma	20	10.0
	Bachelor’s	24	12.0
Occupation	Employed	36	18.0
	Housewife/unemployed	156	78.0
	Retired	8	4.0
Cancer type	Lung	32	16.0
	Prostate	26	13.0
	Breast	26	13.0
	Gastrointestinal tract (GIT)	26	13.0
	Gastric	14	7.0
	Colon	22	11.0
	Other ^1^	54	27.0
Cancer stage (AJCC)	Stage I	38	19.0
	Stage II	98	49.0
	Stage III	56	28.0
	Stage IV	8	4.0
Treatment modality	Not currently receiving treatment	12	6.0
	Chemotherapy (mono)	92	46.0
	Combination therapy ^2^	80	40.0
	Radiotherapy (mono)	12	6.0
	Surgery (mono)	4	2.0
Chemotherapy duration	Mean (SD): 11.56 ± 10.24 months		
Total treatment duration	Mean (SD): 17.31 ± 14.98 months		

^1^ “Other” cancer types include head and neck, lymphoma, ovarian, cervical, brain, and other less frequent solid tumor diagnoses. ^2^ Combination therapy denotes the concurrent or sequential use of two or more modalities (most commonly chemotherapy + radiotherapy or chemotherapy + surgery). AJCC, American Joint Committee on Cancer; GIT, gastrointestinal tract.

**Table 2:** Psychological distress and HRQoL scores by AJCC stage.

Outcome	Stage	*n* (%)	Mean (SD)	*p*-Value
Anxiety (HADS-A)	I	38 (19.0)	6.2 ± 2.8	<0.001
	II	98 (49.0)	9.5 ± 3.2	
	III	56 (28.0)	12.8 ± 3.5	
	IV	8 (4.0)	16.5 ± 3.9	
Depression (HADS-D)	I	38 (19.0)	5.8 ± 2.5	<0.001
	II	98 (49.0)	8.9 ± 3.1	
	III	56 (28.0)	13.1 ± 3.7	
	IV	8 (4.0)	17.2 ± 4.1	
HRQoL (FACT-G)	I	38 (19.0)	82.5 ± 10.2	<0.001
	II	98 (49.0)	74.3 ± 11.8	
	III	56 (28.0)	62.4 ± 12.5	
	IV	8 (4.0)	48.9 ± 13.8	

HADS-A: Hospital Anxiety and Depression Scale–Anxiety subscale; HADS-D: Depression subscale; FACT-G: Functional Assessment of Cancer Therapy–General (total score). *p*-values derived from one-way ANOVA; Tukey HSD post hoc tests indicated significant differences (*p* < 0.05) between Stages III and IV and Stages I–II for all outcomes.

**Table 3:** Sociodemographic and clinical correlates of anxiety severity (HADS-A; panel **A**) and depression severity (HADS-D; panel **B**).

(**A**)						
**Variable**	**Category**	**Total *n***	**Non-Severe (0–15), *n* (%)**	**Severe (16–21), *n* (%)**	***p*-Value**	**Test**
Age, years	<30	42	14 (33.3)	28 (66.7)	**<0.001**	Pearson χ^2^
	30–44	50	28 (56.0)	22 (44.0)		
	45–59	68	48 (70.6)	20 (29.4)		
	≥60	40	28 (70.0)	12 (30.0)		
Sex	Male	128	88 (68.8)	40 (31.2)	**<0.001**	Pearson χ^2^
	Female	72	30 (41.7)	42 (58.3)		
Marital status	Single	40	12 (30.0)	28 (70.0)	**<0.001**	Exact/Monte Carlo
	Married	124	86 (69.4)	38 (30.6)		
	Widowed	12	8 (66.7)	4 (33.3)		
	Divorced	24	12 (50.0)	12 (50.0)		
Cancer stage	I	38	22 (57.9)	16 (42.1)	0.626	Exact/Monte Carlo
	II	98	62 (63.3)	36 (36.7)		
	III	56	30 (53.6)	26 (46.4)		
	IV	8	4 (50.0)	4 (50.0)		
Treatment modality	None	12	2 (16.7)	10 (83.3)	**0.002**	Exact/Monte Carlo
	Chemotherapy	92	60 (65.2)	32 (34.8)		
	Combination therapy	80	48 (60.0)	32 (40.0)		
	Radiotherapy	12	8 (66.7)	4 (33.3)		
	Surgery	4	0 (0.0)	4 (100.0)		
(**B**)						
**Variable**	**Category**	**Total *n***	**Non-Severe (0–15), *n* (%)**	**Severe (16–21), *n* (%)**	***p*** **-Value**	**Test**
Age, years	<30	42	20 (47.6)	22 (52.4)	**0.005**	Pearson χ^2^
	30–44	50	30 (60.0)	20 (40.0)		
	45–59	68	54 (79.4)	14 (20.6)		
	≥60	40	28 (70.0)	12 (30.0)		
Sex	Male	128	96 (75.0)	32 (25.0)	**<0.001**	Pearson χ^2^
	Female	72	36 (50.0)	36 (50.0)		
Marital status	Single	40	20 (50.0)	20 (50.0)	**<0.001**	Exact/Monte Carlo
	Married	124	100 (80.6)	24 (19.4)		
	Widowed	12	4 (33.3)	8 (66.7)		
	Divorced	24	8 (33.3)	16 (66.7)		
Cancer stage	I	38	26 (68.4)	12 (31.6)	0.223	Exact/Monte Carlo
	II	98	70 (71.4)	28 (28.6)		
	III	56	32 (57.1)	24 (42.9)		
	IV	8	4 (50.0)	4 (50.0)		
Treatment modality	None	12	6 (50.0)	6 (50.0)	0.530	Exact/Monte Carlo
	Chemotherapy	92	62 (67.4)	30 (32.6)		
	Combination therapy	80	52 (65.0)	28 (35.0)		
	Radiotherapy	12	8 (66.7)	4 (33.3)		
	Surgery	4	4 (100.0)	0 (0.0)		

Values are presented as observed *n* and row percentages. Pearson’s chi-square test was used where its assumptions were satisfied. For sparse contingency tables, the Fisher–Freeman–Halton exact test was estimated using Monte Carlo simulation with 1,000,000 samples. Bold *p*-values indicate statistical significance at *p* < 0.05.

**Table 4:** Pearson correlations among HADS subscales and FACT-G total score.

Variable	HADS-A	HADS-D	FACT-G
HADS-A	1.000		
HADS-D	0.741 ***	1.000	
FACT-G (total)	−0.750 ***	−0.780 ***	1.000

HADS-A: Hospital Anxiety and Depression Scale, anxiety subscale; HADS-D: depression subscale; FACT-G: Functional Assessment of Cancer Therapy–General (total score). *** *p* < 0.001.

**Table 5:** Multiple linear regression model predicting FACT-G total score (HRQoL).

Predictor	B	SE	β	t	*p*	95% CI	VIF95.
(Constant)	102.45	4.82	—	21.26	<0.001	[93.0, 111.9]	—
HADS-A	−1.12	0.18	−0.34	−6.22	<0.001	[−1.47, −0.77]	2.58
HADS-D	−1.35	0.19	−0.41	−7.11	<0.001	[−1.72, −0.98]	2.61
Age (years)	−0.08	0.06	−0.07	−1.33	0.185	[−0.19, 0.04]	1.12
Sex (Male)	−2.15	1.52	−0.07	−1.41	0.160	[−5.15, 0.85]	1.18
Marital status (Married)	−1.85	1.21	−0.08	−1.53	0.128	[−4.23, 0.53]	1.29
Cancer stage (AJCC)	−1.98	0.89	−0.11	−2.22	0.028	[−3.74, −0.22]	1.15
Treatment modality (Chemotherapy)	0.73	1.05	0.04	0.70	0.487	[−1.34, 2.80]	1.22

Model summary: R^2^ = 0.62; Adjusted R^2^ = 0.60; F (7, 192) = 41.8, *p* < 0.001; B = unstandardized coefficient; SE = standard error; β = standardized coefficient; CI = confidence interval; VIF = variance inflation factor. HADS-A: Hospital Anxiety and Depression Scale, Anxiety subscale; HADS-D: Depression subscale. All VIF values were <3, indicating no multicollinearity.

## Data Availability

The de-identified datasets generated and analyzed during the current study are available from the corresponding author upon reasonable request. The data are not publicly available because of the privacy and ethical restrictions imposed by the participating institutions.

## Abbreviations

The following abbreviations are used in this manuscript.

AJCC	American Joint Committee on Cancer
ANOVA	Analysis of Variance
EWB	Emotional Well-Being (FACT-G subscale)
FACT-G	Functional Assessment of Cancer Therapy–General
FWB	Functional Well-Being (FACT-G subscale)
GenAI	Generative Artificial Intelligence
GIT	Gastrointestinal Tract
HADS	Hospital Anxiety and Depression Scale
HADS-A	Hospital Anxiety and Depression Scale, Anxiety subscale
HADS-D	Hospital Anxiety and Depression Scale, Depression subscale
HRQoL	Health-Related Quality of Life
HSD	Honestly Significant Difference (Tukey’s post hoc test)
IRB	Institutional Review Board
PWB	Physical Well-Being (FACT-G subscale)
QoL	Quality of Life
SD	Standard Deviation
SPSS	Statistical Package for the Social Sciences
SWB	Social/Family Well-Being (FACT-G subscale)
TNM	Tumour, Node, Metastasis (staging classification)
VIF	Variance Inflation Factor
